# Application of Nanostructures in Biology and Medicine

**DOI:** 10.3390/ijms25189931

**Published:** 2024-09-14

**Authors:** Kirill Lozovoy

**Affiliations:** Department of Quantum Electronics and Photonics, Faculty of Radiophysics, National Research Tomsk State University, Lenin Av. 36, 634050 Tomsk, Russia; lozovoymailbox@gmail.com

At present, nanomaterials are used in a wide range of applications in all spheres of civil needs, including energy, medicine, and industry [1,2]. Moreover, they are considered one of the most promising classes of materials for the next generation of technological development [3,4,5]. The interest in the possibilities of nanoparticles and nanodevices allows for deeper study of the physical properties of these new materials and provides a starting point for the development of a huge number of practically important areas, from synthetic biology, drug delivery platforms [6,7,8,9,10], and brain–computer interfaces [11,12,13,14,15] to nanoelectronics [16,17,18], nanophotonics [19,20,21,22,23], and quantum communications technologies [24,25].

This Special Issue focuses on recent research in various fields of applied nanoscience, including materials science, chemistry, molecular and cell biology, and biotechnology. Special attention is paid to the state-of-the-art methods for synthesizing and characterizing advanced materials, nanoparticles, and biological objects, as well as their emerging applications.

This Special Issue starts with a comprehensive review article by da Silva et al. [26], considering the full spectrum of the use of microparticles in pharmaceutics. Microparticles are any particles with a size of 1–1000 µm (Figure 1). They are widely used as drug delivery systems because they offer superior therapeutic and diagnostic performance compared to conventional modes of drug delivery. This review focuses on the contemporary in vivo and in vitro applications of different active pharmaceutical ingredients microencapsulated in polymeric or lipid matrices, discussing the potential applicability of microparticulate systems in the pharmaceutical field.

This general review is followed by a more narrowly focused work by Urbano-Gámez et al. [27], addressing important questions concerning cancer therapy with nanoparticles. The application of metal-based nanoparticles in cancer therapy and diagnostics (theranostics) [28,29] has been a hot research topic since the early days of nanotechnology and has become even more relevant in recent years [30,31]. In this review, a critical analysis of key challenges that must be addressed for the successful targeting of either tumor tissue or cancer cells within the tumor tissue is carried out (Figure 2).

Kah Sem et al. [32] work in an adjacent field, dealing with the use of nanoparticles to combat animal diseases. Their review is based on studies on vibriosis, one of the most common diseases in marine aquaculture, affecting many species of economically significant aquatic organisms around the world [33,34]. The use of graphene oxide and nanoparticles in the treatment of vibriosis is explored in this article.

Tsilo et al. [35] study a slightly different area: the use of nanoparticles in wastewater treatment, another urgent problem [36]. Their study utilized Fe nanoparticles that were synthesized using a bioflocculant to eliminate different kinds of pollutants and dyes found in wastewater and solutions.

Zhao et al. [37] study the chemical applications of mesoporous nanomaterials. In their study, ionic magnetic mesoporous nanomaterials with high absorptivity for ethanol amines and cyanide were successfully synthesized. The potential of these materials in the verification of chemical weapons and the destruction of toxic chemicals was shown.

Finally, in an original work by Lepekhina et al. [38], a new approach to assessing cell viability based on two-photon microscopy is described. The study of cell viability is included in the list of mandatory studies when creating new materials for implants intended to replace hard tissues [39,40]. In this way, the biocompatibility of implants with the human body is assessed. Scientists from Tomsk State University have developed a method that allows for the real-time determination of the state of the cells as an indicator of implant survival. The fluorescence lifetime imaging microscopy (FLIM) results obtained in this work can be used as additional information for scientists who are interested in manufacturing osteoimplants. This new approach will make it possible to create materials with high biocompatibility for reconstructive surgery and, accordingly, improve the quality of life of patients.

## Figures and Tables

**Figure 1:**
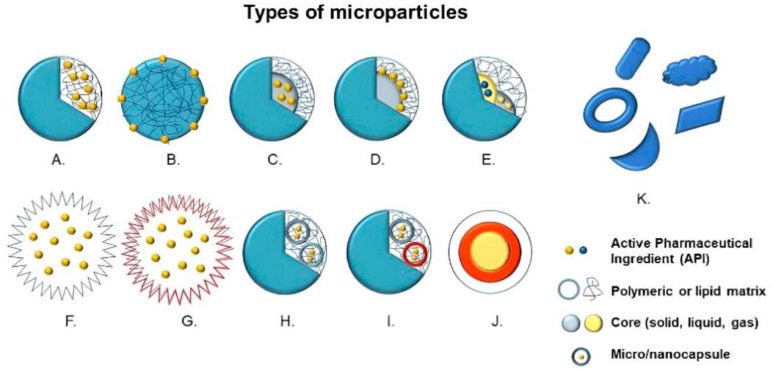
Types of microparticles: (**A**) microsphere with entrapped active pharmaceutical ingredient (API); (**B**) microsphere with adsorbed API; (**C**) microcapsule with entrapped API; (**D**) microcapsule with adsorbed API; (**E**) multinucleated microcapsule; (**F**) hollow microparticle; (**G**) hollow microparticle with several layers; (**H**) microparticle containing microcapsules; (**I**) microparticle containing multinucleated microcapsules; (**J**) multilayer microparticles; and (**K**) microparticles with irregular shapes [26].

**Figure 2:**
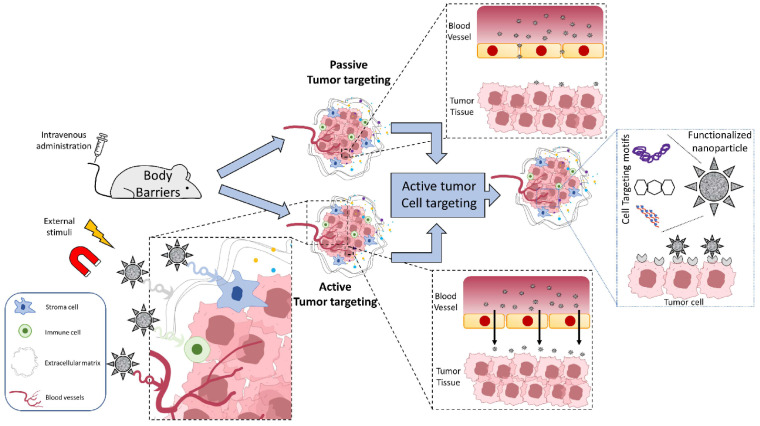
Tumor targeting (passive or active) and tumor cell targeting [27].
